# Feeding and swallowing in cerebral palsy: Caregiver burden, quality of life and support needs

**DOI:** 10.4102/sajcd.v72i2.1124

**Published:** 2025-11-21

**Authors:** Raquel Lewis, Michal Harty, Vivienne Norman

**Affiliations:** 1 Division of Communication Sciences and Disorders, Department of Health and Rehabilitation Sciences, Faculty of Health Sciences, University of Cape Town, Cape Town, South Africa

**Keywords:** cerebral palsy, caregiver burden, feeding difficulties, swallowing difficulties, quality of life, support needs, South Africa, low- and middle-income countries

## Abstract

**Background:**

Children with cerebral palsy (CP) often experience feeding and/or swallowing difficulties (FSD), which are lifelong, necessitating long-term care by caregivers, particularly in resource-constrained contexts like South Africa. This ongoing responsibility can lead to burden and reduced quality of life (QoL) for the caregiver and child. Exploring caregivers’ experiences is required for planning and implementation of effective support services.

**Objectives:**

The study aimed to explore the experiences of caregivers of children with CP who have FSD, regarding the burden, QoL and support needs.

**Method:**

A qualitative case study design was used, involving eight mothers of children aged 2–8 years, recruited from a hospital-based CP clinic. Participants (aged 25–42 years) engaged in semi-structured telephonic interviews conducted in English and isiXhosa. Thematic analysis was conducted to generate themes and sub-themes.

**Results:**

Thematic analysis yielded seven main themes: *Worry*; *Feeding is everything*; *Identified support needs*; *What helps me cope?*; *Cost of caregiving*; *Hopeful caregiving* and *Shortfalls of healthcare system and society*, reflecting emotional strain, the centrality of feeding, specific support requirements, caregiver resilience, multifaceted burden and hope.

**Conclusion:**

Caregiver burden and QoL are closely linked and shaped by the emotional, physical, social and financial demands of caregiving. While caregivers described their unmet support needs, they also identified positive aspects of caregiving, including a sense of purpose and connection with their child.

**Contribution:**

The findings offer insight into caregiver experiences in South Africa and underscore the need for tailored, contextually relevant interventions to improve caregiver well-being and child outcomes.

## Introduction

Advances in global child health have led to reductions in child mortality. However, they have also contributed to a rising prevalence of childhood disability, particularly in low- and middle-income countries (LMICs) (Nannan et al., 2019). Cerebral palsy (CP), a non-progressive disorder of movement and posture caused by early brain damage (Bax et al., 2005), remains the most common cause of childhood disability worldwide (Oskoui et al., 2013). In South Africa, estimates suggest a higher prevalence than in high-income countries (Maphumulo & Bhengu, 2019).

Conventional healthcare responses to CP are shaped by the biomedical model, focusing narrowly on physical impairments (Leite et al., 2021). In contrast, social and family-centred models, such as the World Health Organization’s (WHO) International Classification of Functioning, Disability and Health (ICF), advocate for broader, context-sensitive approaches that consider the environmental and familial impact of disability (Abdel Malek et al., 2020). Cerebral palsy is a lifelong disability requiring healthcare resources and continuing care (Sewell et al., 2014). In many cases, children with CP experience associated impairments, such as feeding and/or swallowing difficulties (FSD). These difficulties are prevalent in children with CP, ranging between 37% and 99%, with increased risk in those with severe motor impairment (Goday et al., 2019; Speyer et al., 2019). The consequences of FSD include malnutrition, dehydration, aspiration pneumonia, respiratory complications and increased hospitalisation risk (Blackmore et al., 2018; Damrongmanee et al., 2021), as well as disruptions to family dynamics, increased caregiver stress and a potentially negative impact on both the child’s and caregiver’s quality of life (QoL) (Greer et al., 2008; Lefton-Greif et al., 2024; Liu et al., 2020).

Feeding and/or swallowing difficulties significantly heighten caregiver burden by complicating basic caregiving tasks and increasing psychological, emotional, physical, social and financial strain (Davis et al., 2010; Sewell et al., 2014), resulting in negative health and QoL consequences. For example, caregivers are advised by healthcare professionals to incorporate modified diets, such as thickening feeds, or feeding techniques, such as positional changes and lateral feeding, to ensure feeding and swallowing safety. Such modifications and techniques require effort and preparation and are generally time consuming and challenging to put into practice (Davis et al., 2010). In addition, the financial cost of specialised foods, supplements, and assistive devices may exhaust the caregiver’s financial resources (Okada et al., 2022). Feeding-related responsibilities further limit caregivers’ engagement in social or professional activities and often lead to internalised stigma and feelings of guilt, especially when non-oral feeding methods such as gastrostomy are introduced (Morrow et al., 2008; Petersen et al., 2006). The demands and responsibilities associated with FSD are all-encompassing for caregivers, who are simultaneously balancing other personal, family and professional obligations (Murphy et al., 2007).

Caregivers assume intense home-based care responsibilities (Davis et al., 2010; Pretorius & Steadman, 2017), and the caregiver’s QoL may be significantly compromised by the cumulative effect of caregiving demands, emotional stress and limited social participation (Polack et al., 2018). The quality of caregiver relationships with partners, extended family, peers and healthcare professionals has a marked influence on coping and self-perception (Hewetson & Singh, 2009; Morrow et al., 2008). Yet, in many cases, caregivers report feeling disempowered by interactions with health professionals, who may discount caregivers’ knowledge or reinforce shame (Hartley et al., 2005). While some caregivers find personal growth, advocacy skills and emotional resilience through caregiving (Bailey & Harrist, 2017), overall caregiver QoL remains fragile, especially in resource-constrained settings where support structures are limited or inaccessible (Pretorius & Steadman, 2017).

The caregiving experience in LMICs is mostly understood based on insights derived from studies in high-income countries. However, many challenges and stressors are unique to caregivers in LMICs (Lefton-Greif et al., 2024). Structural barriers such as food scarcity, inaccessible transportation and insufficient rehabilitation services in LMICs exacerbate the burden on caregivers (Dambi et al., 2015; Thrush & Hyder, 2014). A study examining the caregiving experience of mothers of children with CP in Zimbabwe, identified that the physical burden of caregiving is exacerbated in such contexts because of the lack of adapted transport services and assistive devices (Dambi et al., 2015). Consequently, mothers resort to the conventional method of carrying their child on their back for transporting and transferring purposes, raising the risk of back pain and injury. Thus, caregivers in LMICs face a significant and neglected burden in physical, psychological, social, financial and time domains (Thrush & Hyder, 2014).

Well-structured, multicomponent psychosocial interventions enhance caregiver competence, self-efficacy and coping capacity (Reinhard et al., 2008). However, access is frequently hindered in LMICs because of infrastructural, linguistic, financial and sociocultural barriers (Pretorius & Steadman, 2017). Peer-support and community-based programmes – such as South Africa’s *Malamulele Onward* and Uganda’s *Akwenda* CP programme – have emerged as locally grounded solutions with the potential to improve caregiver QoL and knowledge, particularly around feeding practices (Saloojee et al., 2021; Zuurmond et al., 2019). Complementary online and telephonic interventions also hold promise for increasing accessibility, though technological limitations in LMICs may be challenging (Taylor et al., 2021). Social networks and respite services are valuable protective factors against caregiver burnout yet are limited in LMICs (Moriwaki et al., 2022; Pretorius & Steadman, 2017). Financial aid (e.g., care dependency grants) is often inadequate or inaccessible, further exacerbating caregiver burden in LMICs (Vadivelan et al., 2020).

Given that caregiver well-being is closely linked to the quality of care and overall well-being of the child, a nuanced understanding of caregiver burden – particularly in relation to FSD – is essential for developing contextually appropriate and caregiver-sensitive interventions (Lefton-Greif et al., 2024). Caregiver perspectives, particularly in resource-constrained settings, remain underexplored despite being central to managing FSD and ensuring successful feeding interventions (Dambi et al., 2015; Pretorius & Steadman, 2017). This study therefore sought to address this gap by exploring caregivers’ experiences of caregiver burden, QoL and support needs in caring for a child with CP who has FSD within the context of the Western Cape, South Africa.

## Research methods and design

### Study design

A qualitative, descriptive case study design was employed to explore caregivers’ experiences, providing a nuanced, context-specific understanding of their lived experiences that may be less accessible through quantitative approaches (Flick, 2018). This design enabled rich, detailed accounts within a defined setting.

### Study population and sampling strategy

The ‘case’ involved eight caregivers of children with CP attending an established CP clinic service within the Cape Metropole of Cape Town, South Africa. Participants were the primary caregivers of children (aged 0–12 years) with CP and FSD. Purposive and convenience sampling were used to recruit information-rich participants relevant to the study’s aim. Because of coronavirus disease 2019 (COVID-19) restrictions, potential participants were identified by the resident speech-language therapist, and with their permission, contacted telephonically by the researcher, with the assistance of the research assistant who acted as an isiXhosa interpreter where necessary. Information sessions were conducted telephonically in participants’ preferred language. Thereafter, potential participants were sent a photograph of the consent script for review, as not all had access to email. Once the participants were satisfied with the information provided and had had their questions answered, those who were willing to participate provided consent, which was recorded. A convenient time was scheduled for the interview.

### Description of participants

All participants were mothers aged 25–42 years (mean = 29.25), caring for children aged 2–8 years (mean = 4.63). Six children were male. Feeding methods varied: five oral, one percutaneous endoscopic gastrostomy (PEG) and two mixed oral and PEG. Participant demographics are summarised in Table 1.

**TABLE 1:** Demographic description of participants.

Participant†	Language	Age (years)	Marital status	Child			Type of feeding
				Relationship to the child	Age (years)	Sex	
1	isiXhosa	27	Single	Mother	5	Male	Oral
2	English	30	Married	Mother	2	Female	Oral
3	English	25	Married	Mother	5	Male	Oral and PEG
4	English	29	Married	Mother	6	Male	Oral and PEG
5	isiXhosa	25	Single	Mother	4	Female	PEG
6	isiXhosa	28	Single	Mother	8	Male	Oral
7	English	42	Married	Mother	3	Male	Oral
8	isiXhosa	28	Single	Mother	4	Male	Oral

*Source:* Le Roux, R.S. (2023). *Caregivers’ perceptions of caregiver burden, quality of life and support needs in caring for a child with cerebral palsy with feeding and/or swallowing difficulties within the context of the Western Cape, South Africa*. Masters dissertation, Department of Health and Rehabilitation Sciences, Faculty of Health Sciences, University of Town. Retrieved from http://hdl.handle.net/11427/39223

PEG, percutaneous endoscopic gastrostomy.

† , Numerical codes assigned for anonymity.

### Data collection

An interview guide was developed using seven open-ended questions, aligned with Krueger’s (2015) structure and informed by the *Feeding/Swallowing Impact Survey* (Lefton-Greif et al., 2014). Four interviews were conducted in English by the researcher and four in isiXhosa by the assistant, with the researcher present. Disruptions because of signal loss were managed with summary prompts upon reconnection. Interviews were audio-recorded for transcription. Summaries of the interview data were confirmed with participants at the end of each interview.

### Data analysis

Thematic analysis was employed to identify and interpret patterns within the data, enabling the development of key themes and sub-themes related to caregivers’ experiences (Gray, 2018). Audio-recorded interviews were anonymised and transcribed by the researcher and isiXhosa assistant, who also translated isiXhosa transcripts into English. The researcher engaged in data familiarisation through repeated reading (Flick, 2018; Gray, 2018). Transcripts were independently reviewed by the researcher and supervisor for accuracy. Manual coding was undertaken by the researcher using colour highlighting to identify emerging ideas. Related codes were grouped into meaning units, then organised into sub-themes and themes. The researcher and supervisor collaboratively mapped codes into thematically coherent categories, with supporting participant quotations tabulated to enhance trustworthiness. Data and themes were revisited to ensure accuracy and faithful representation of caregivers’ experiences. Themes were reviewed by the research co-supervisor, independent of the coding process, to confirm alignment with the data and core concepts (Gray, 2018). Themes were refined and named to reflect their conceptual meaning.

Trustworthiness was established through credibility, transferability and confirmability (Patton, 2015). Credibility was supported by pilot testing the interview guide and informal member checking during interview summaries (Gray, 2018). Interviews were transcribed verbatim and independently reviewed by the researcher and supervisor. Analyst triangulation and advocacy–adversary analysis (Patton, 2015) were used to enhance credibility through collaborative theme validation. Data source triangulation through cross-checking the consistency of data across interviews and frequent debriefing sessions helped mitigate bias and refine interpretations (Flick, 2018). A consistent set of interview questions supported transferability. While the findings are context-specific, the detailed methodology enables replication in other settings. Finally, reflexivity was maintained via a journal and regular supervisory meetings to reflect on positionality and potential bias (Flick, 2018). Audio recordings and direct quotes ensured that findings were supported by participant data.

### Ethical considerations

Ethics approval was granted on 02 April 2020 by the University of Cape Town Faculty of Health Sciences’ Human Research Ethics Committee (HREC 097/2020), with specific amendments to adhere to COVID-19 regulations and restrictions. The study was registered with the National Health Research Database (WC_202009_056), and permission was obtained from the research site. The study adhered to the ethical principles outlined in the Declaration of Helsinki (World Medical Association [WMA], 2025). Ethical research conduct was ensured by upholding the principles of autonomy through informed consent; confidentiality and anonymity by using participant codes and removing identifying information; non-maleficence and beneficence – there was no risk of physical harm to the participants and counselling support was available on site if needed and justice in equal and fair opportunity for inclusion in the study.

## Results

**FIGURE 1:**
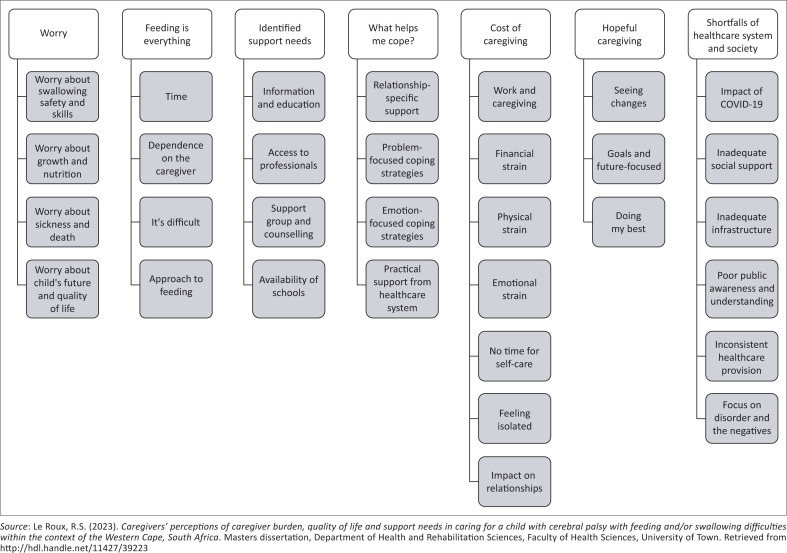
Main themes and sub-themes.

Seven main themes emerged from the data analysis: *Worry, Feeding is everything, Identified support needs, What helps me cope?, Cost of caregiving, Hopeful caregiving* and *Shortfalls of the healthcare system and society* (Le Roux, 2023). These themes, along with their corresponding sub-themes, are presented in Figure 1.

### Theme 1: Worry

Caring for a child with CP and FSD generates significant worry for mothers. Key concerns included swallowing safety, growth, nutrition, sickness, death and the child’s future QoL. Caregivers expressed heightened concern over feeding, particularly the risk of choking (Table 2).

**TABLE 2:** Theme 1: *Worry* – participant quotes.

Sub-theme	Quote
Worry about swallowing safety and skills	‘I worry that … when I’m busy feeding her … something gets stuck … in her throat and she chokes and I dunno how to help bring it out.’ (Participant 2) ‘I don’t try too much textured foods because I’m afraid she can’t swallow fast enough or … she’ll choke and you know, something can go wrong.’ (Participant 2)
	‘My only concern is when I feed him something for the first time, like “Is the child going to choke on it?”, “Is it smooth enough for him?”, “Will he be able to chew it?”’ (Participant 7)
Worry about growth and nutrition	‘He doesn’t get enough nutrition because he doesn’t eat food. He lives on milk always.’ (Participant 5)
	‘I’m worrying about his weight. He’s not gaining but he eat a lot of food.’ (Participant 4)
Worry about sickness and death	‘When he refluxes on the food and when it goes into his lungs … then it worries me a bit because then he gets sick and that is when he ends up in hospital.’ (Participant 3)
	‘I always had this worry that she would um pass on. That was my biggest worry.’ (Participant 2)
Worry about child’s future and quality of life	‘My concern is, since he is this way, will he go to school or what will he do because he can’t speak or walk?’ (Participant 5)
	‘My worries about the future, really, is that um if I’m not gonna be one day with him who’s gonna look after him? And will the next person have the patience that I have with him and will he be able to feed himself and do stuff for himself?’ (Participant 7)

*Source*: Le Roux, R.S. (2023). *Caregivers’ perceptions of caregiver burden, quality of life and support needs in caring for a child with cerebral palsy with feeding and/or swallowing difficulties within the context of the Western Cape, South Africa*. Masters dissertation, Department of Health and Rehabilitation Sciences, Faculty of Health Sciences, University of Town. Retrieved from http://hdl.handle.net/11427/39223

### Theme 2: Feeding is everything

Caregivers underscored the dominant role of feeding in their lives, noting its time-consuming nature, the child’s dependence and the emotional and physical demands of feeding tasks (Table 3).

**TABLE 3:** Theme 2: *Feeding is everything* – participant quotes.

Sub-theme	Quote
Time	‘It definitely takes longer [*compared to feeding a sibling*].’ (Participant 3)
	‘He needs his food to be prepared separately. It’s a struggle, because we can’t feed him food like ours and he can’t chew.’ (Participant 6)
	‘You must be considerate … it takes a while to feed.’ (Participant 8)
Dependence on the caregiver	‘[*I*] won’t leave him by someone else cos I know it’s [*feeding*] gonna be a struggle …’ (Participant 3)
	‘Not everyone can feed him … It’s only me that feeds.’ (Participant 7)
	‘It will always just be me feeding her because I wouldn’t trust … anyone else to feed her … I’m scared that they can tell me that she don’t wanna eat or that she’s not hungry but to me that is you not doing it properly.’ (Participant 2)
It’s difficult	‘I must … feed him on my lap and sometimes, he don’t wanna sit up straight, just wanna lay [*on his*] back. It’s … very difficult.’ (Participant 7)
	‘He moves. It’s a struggle. He removes the PEG some days. It’s very difficult.’ (Participant 5)
Approach to feeding	‘You just have to be patient … this may take some time um so you sit in a position where you’re comfortable and you feed her.’ (Participant 2)
	‘You shouldn’t rush because the child chokes and he can die.’ (Participant 8)

*Source*: Le Roux, R.S. (2023). *Caregivers’ perceptions of caregiver burden, quality of life and support needs in caring for a child with cerebral palsy with feeding and/or swallowing difficulties within the context of the Western Cape, South Africa*. Masters dissertation, Department of Health and Rehabilitation Sciences, Faculty of Health Sciences, University of Town. Retrieved from http://hdl.handle.net/11427/39223

### Theme 3: Identified support needs

Caregivers identified several unmet support needs that, if met, could reduce caregiver burden and improve QoL. The caregivers emphasised the need for clearer information and guidance from healthcare professionals, regular professional contact and reassurance and social support. Table 4 summarises the relevant subthemes.

**TABLE 4:** Theme 3: *Identified support needs* – participant quotes.

Sub-theme	Quote
Information and education	‘We need more information about how to feed our kids.’ (Participant 2)
	‘Maybe they, you know, give a document to the caregiver as well, just to take it with and try and implement it; what you need to do … maybe practical and us physically doing it and making sure that we’re doing it right.’ (Participant 2)
Access to professionals	‘He needs to see his doctors often. The small stuff is the main stuff that is needed for support.’ (Participant 3)
	‘Yeah, someone that you can send a message to … this is what’s happening, what should I do? We all have Whatsapp you know. It’s just easier … sometimes different situations happening at different times and you’re not always sure what to do.’ (Participant 2)
Support group and counselling	‘Meet mothers who have children with CP, yes. We can meet and talk, maybe form a support group.’ (Participant 8)
	‘[*A support group*] will be very good for me … then it will feel that someone else will understand what I’m going through and how when I basically say ‘try this’ but they say ‘try that’ … but instead I experience everything else on my own.’ (Participant 7)
Availability of schools	‘There aren’t like a lot of schools available for children … to be put in a good school that can take care of them.’ (Participant 2)

*Source*: Le Roux, R.S. (2023). *Caregivers’ perceptions of caregiver burden, quality of life and support needs in caring for a child with cerebral palsy with feeding and/or swallowing difficulties within the context of the Western Cape, South Africa*. Masters dissertation, Department of Health and Rehabilitation Sciences, Faculty of Health Sciences, University of Town. Retrieved from http://hdl.handle.net/11427/39223

### Theme 4: What helps me cope?

Despite unmet needs, caregivers were able to identify several available intrinsic and extrinsic supports that facilitated their capacity to cope. A social support network, self-taught coping strategies, religion and spirituality and practical support from the healthcare system were the most posited coping facilitators. Although participants identified a lack of mother-to-mother support, relationship-specific support from family, friends, neighbours, healthcare professionals and their community was valued and provided relief for caregivers (Table 5).

**TABLE 5:** Theme 4: *What helps me cope?* – participant quotes.

Sub-theme	Quote
Relationship-specific support	‘For example, from friends, family and neighbours … if my mother is not around, they watch him for me, perhaps I must go somewhere.’ (Participant 6)
	‘No, at home they love my child a lot. They support me a lot. Everyone supports me. No one is embarrassed. Whoever wants to go out with him they do.’ (Participant 8)
Problem-focused coping strategies	‘I take my notebook and I take notes and I take pictures with my phone.’ (Participant 2)
	‘So what I do is they show me something then I try it out but I try to do what is most comfortable for me and what is the best way for me and for him … I ask my husband to make a video or something then I’ll just go show it to them. What do they think, is it a good idea or what do they think I’m not doing it right, stuff like that.’ (Participant 7)
Emotion-focused coping strategies	‘I have a lot of faith in God … I trust that like even if I tried 10 million times like and it doesn’t work … It’s because God doesn’t want it to work.’ (Participant 2)
	‘Writing the whole process now because I just want to read it and just let go.’ (Participant 2)
Practical support from healthcare system	‘The clinic helping me with the supply of his milk … before I got the supply of his milk, he was just getting three or four tins a year.’ (Participant 3)
	‘Having the dietician and the speech therapist working together, they give me quite a bit of information. So, the dietitian will adjust his meals and then the speech therapist will show me how to deal with feeding him.’ (Participant 3)

*Source*: Le Roux, R.S. (2023). *Caregivers’ perceptions of caregiver burden, quality of life and support needs in caring for a child with cerebral palsy with feeding and/or swallowing difficulties within the context of the Western Cape, South Africa*. Masters dissertation, Department of Health and Rehabilitation Sciences, Faculty of Health Sciences, University of Town. Retrieved from http://hdl.handle.net/11427/39223

### Theme 5: Cost of caregiving

The ‘cost of caregiving’ included financial, emotional and social sacrifices, with caregivers detailing the impact of being a caregiver of a child with CP and chronic FSD on their employment status, financial stability, physical and emotional health and social participation. Table 6 outlines the associated subthemes and illustrative quotations for this theme.

**TABLE 6:** Theme 5: *Cost of caregiving* – participant quotes.

Sub-theme	Quote
Work and caregiving	‘I’ll give up my job. It wasn’t an easy decision for me to make because I still wanted to work and be independent.’ (Participant 2)
	‘Yes, I miss it because I was working for 16 years and it was very difficult in the beginning.’ (Participant 7)
Financial strain	‘It’s still difficult actually, now, financially, because I was the only one that was working and, yeah, it’s like such a strain on me and pressure. Like sometimes, there’s not enough money for, you know, food, clothing, stuff like that.’ (Participant 7)
	‘It’s expensive. I don’t have the money …’ (Participant 8)
Physical strain	‘He is heavy … I always have back pain.’ (Participant 6)
	‘It is quite hard because sitting in that position for such a long time cos he takes so long to eat as well. And it can get to my arms and my back, and the job that I’m also doing it’s also like straining on my body, so I’m straining at home and at work.’ (Participant 3)
Emotional strain	‘The child’s life stands still and you see his peer starting with crèche but he is doing nothing and there is no hope that he will. I ended up with depression. I need a lot of counselling.’ (Participant 8)
	‘I don’t know if I actually really dealt with … giving birth to a different child and [*I haven’t*] really spoken about it … the process.’ (Participant 2)
No time for self-care	‘I must feed him five times a day … I only have [*a*] meal one time a day … Sometimes I must wait ‘til nine o’clock at night to wash me. I had to cut off my hair because I didn’t have time to do my hair. You see so basically to plan my life because everything revolves around him.’ (Participant 7)
Feeling isolated	‘I don’t actually feel now comfortable going to social places … withdraw myself, like going out or be back late or stuff like that.’ (Participant 7)
	‘I once went to church. He cried so much I haven’t gone again.’ (Participant 5)
Impact on relationships	‘It’s just about him, like nothing else so it … ha[*s*] a big impact on a person’s marriage and relationship with different kind of people and with friends and family members.’ (Participant 7)

*Source*: Le Roux, R.S. (2023). *Caregivers’ perceptions of caregiver burden, quality of life and support needs in caring for a child with cerebral palsy with feeding and/or swallowing difficulties within the context of the Western Cape, South Africa*. Masters dissertation, Department of Health and Rehabilitation Sciences, Faculty of Health Sciences, University of Town. Retrieved from http://hdl.handle.net/11427/39223

### Theme 6: Hopeful caregiving

Despite challenges, caregivers felt a deep sense of hope and connection as they observed positive changes, established realistic goals and maintained a future-focused outlook. Many attributed the positive changes in their child and their relationship to their caregiving efforts (Table 7).

**TABLE 7:** Theme 6: *Hopeful caregiving* – participant quotes.

Sub-theme	Quote
Seeing changes	‘As long as she seems happy, that is like most important to me and … with this one week that I’ve been at home, I feel that I can already see, like, a difference in her.’ (Participant 2)
	‘Him connecting with me and also in a very special way. In a special way that no one else knows and that also makes me feel much better.’ (Participant 3)
Goals and future focused	‘We have to give her the care that she needs so that she can be the best version of herself. Um whether it is that she remains this way and that she seems happy and that she seems like she does have a good quality life um that is … what is important … I think it will be worth it in the end.’ (Participant 2)
Doing my best	‘If we teach our children and we are persistent then maybe they can have, you know, a better quality of life if we just keep on trying and even if it doesn’t work um at least we know that we’ve tried our best.’ (Participant 2)

*Source*: Le Roux, R.S. (2023). *Caregivers’ perceptions of caregiver burden, quality of life and support needs in caring for a child with cerebral palsy with feeding and/or swallowing difficulties within the context of the Western Cape, South Africa*. Masters dissertation, Department of Health and Rehabilitation Sciences, Faculty of Health Sciences, University of Town. Retrieved from http://hdl.handle.net/11427/39223

### Theme 7: Shortfalls of healthcare system and society

The participants noted barriers related to the access and quality of healthcare services exacerbated by the COVID-19 pandemic, social support, infrastructure and a lack of disability awareness (Table 8).

**TABLE 8:** Theme 7: *Shortfalls of healthcare system and society* – participant quotes.

Sub-theme	Quote
Impact of COVID-19	‘We used to do it together, myself and my husband, but because of COVID they only allowed one parent in so I’ve been taking her.’ (Participant 2)
	‘He had a few doctor’s appointments but it was cancelled numerous times because the clinics are not open yet or we couldn’t see anyone because it was just closed … and I can’t just take him to the doctor because he’s also in danger of getting the COVID.’ (Participant 3)
	‘If the pandemic wasn’t here I think I would have like gone to the library or … try to reach out to other mothers with children with the same condition as [*child’s name*] … people don’t want visitors. They don’t want support groups up close and stuff like that. There’s nowhere I can go to seek help at this moment.’ (Participant 7)
Inadequate social support	‘I’m not receiving support because at the hospital they wanted the disability grant forms but since my mother used to receive the disability grant it’s a struggle now to make changes. When we … buy his food, we use the R400 from his child support grant.’ (Participant 5)
Inadequate infrastructure	‘I can’t [*go*] in a taxi with him cos there’s too much noise that goes through the body and it irritates him … So, I need to take Uber with him wherever I go. Uber is quite costly.’ (Participant 3)
Poor public awareness and understanding	‘When I feed him with a tube, people walk past and they stare and they’re like what is this pipe coming out of his top here? Everywhere I go people always has questions to ask … ‘Why you doing that? For what is that?’ They don’t understand.’ (Participant 3)
Inconsistent healthcare provision	‘I noticed that [*different province*] was failing the child. They weren’t giving me anything as if they were saying we should wait for the day ‘til he dies. So I went and searched google for a hospital that can help with CP and found that [*institution’s name*] is number one and it helps with children. So [*institution’s name*] helped a lot. They gave him a chair, medication, and everything.’ (Participant 8)
	‘A therapist was going to call me regarding information about a child with a PEG after he got it but they didn’t contact me.’ (Participant 5)
Focus on disorder and negatives	‘We were actually told that she wasn’t gonna eat normal … those are not the type of things that you want to hear when it’s your child, and the doctors know that there’s a possibility that you can teach her they shouldn’t be telling, you know that, your child won’t eat, you know what I mean?’ (Participant 2)
	‘When you go to the doctor … it’s not a jolly visit because obviously they giving me facts about [*child’s name*] so it’s not always good … Sometimes it’s not bad but there’s never a time that there’s not something bad you know, then it’s always this and his hips and his this and his that and his eyesight …’ (Participant 3)

*Source*: Le Roux, R.S. (2023). *Caregivers’ perceptions of caregiver burden, quality of life and support needs in caring for a child with cerebral palsy with feeding and/or swallowing difficulties within the context of the Western Cape, South Africa*. Masters dissertation, Department of Health and Rehabilitation Sciences, Faculty of Health Sciences, University of Town. Retrieved from http://hdl.handle.net/11427/39223

COVID-19, coronavirus disease 2019; PEG, percutaneous endoscopic gastrostomy.

## Discussion

The study identified several main themes in caregivers’ perceptions of burden, QoL and support. The themes of *Worry, Feeding is everything, Identified support needs, What helps me cope?, Cost of caregiving, Hopeful caregiving* and *Shortfalls of healthcare system and society* demonstrated that caring for a child with CP who also has FSD has a significant impact on the lives of caregivers. The themes and sub-themes highlight the tension between the needs of the child and those of the caregiver, where the child’s well-being often conflicts with that of the caregiver. These findings emphasise the importance of a shift towards a family-centred approach and the use of frameworks like the ICF, which address both the child’s and the caregiver’s needs (Abdel Malek et al., 2020), as caregivers often require care as much as the children they care for (Reinhard et al., 2008).

The study found a mirroring of themes, where certain aspects of caregiving both helped and hindered caregivers’ ability to cope. For example, support from family, friends, community members and healthcare professionals was crucial in coping with caregiving tasks. However, caregivers also experienced loneliness, limited respite and strained relationships. This duality supports the assertion that while social support networks can mitigate caregiver burden and improve QoL (Taylor et al., 2021), caregiving also leads to isolation and reduced QoL (Davis et al., 2010). A similar duality was observed in the support received from the healthcare system. Positive interactions, such as receiving nutritional supplements and specialised seating, alleviated some of the caregivers’ physical and financial burdens. However, negative encounters with inconsistent service provision increased caregivers’ burden and challenges, reflecting the findings of Morrow et al. (2008), where caregivers reported both beneficial and unsatisfactory healthcare experiences. The themes illustrated the multifaceted strain of caregiving, as identified by Liu et al. (2020), encompassing emotional, social, physical and financial stressors. The combination of these challenges underscores the complexity of caregiver burden.

The theme of *Worry* captured the emotional strain caregivers felt regarding feeding-related tasks and potential adverse consequences of FSD. Caregivers’ worry appeared chronic, stemming from concerns of choking, inadequate nutrition and aspiration. These feeding-related anxieties have been recognised in other studies as significant sources of caregiver stress, negatively affecting their QoL (Jones et al., 2018; Polack et al., 2018; Taylor et al., 2021). The challenge of ensuring the child’s safety and well-being while meeting their nutritional needs led to feelings of self-doubt, echoing previous findings where caregivers questioned their competence (Greer et al., 2008; Hewetson & Singh, 2009).

*Feeding is everything* emphasised the centrality of feeding in caregivers’ daily lives, where time and effort were devoted to nourishment, leaving little room for socialisation or work. This aligns with Taylor et al.’s (2021) concept of a ‘child-centred world’, where caregivers’ lives revolve around feeding-related activities. Time constraints were a major concern, with caregivers needing to be patient and focused on their child’s comfort. Notably, the time burden of caregiving was similar regardless of whether the child was fed orally or via a tube, an unexpected finding considering the assumption that tube feeding might be less time consuming. This could be explained by factors such as the time required for training, maintaining the feeding site and addressing the child’s tolerance of the feeds, as proposed by Sleigh (2005).

The *Cost of caregiving* theme underscores the negative impact on caregivers’ well-being, with emotional strain, depression and anxiety frequently reported as a result of the responsibilities of caring for a child with CP and FSD. These feelings, consistent with previous research, correlate with a decline in caregiver QoL (Gugała, 2021). Social strain also emerged as a significant challenge, with caregivers experiencing isolation because of limited opportunities for social participation, which further exacerbated depressive symptoms (Polack et al., 2018; Zuurmond et al., 2019). The physical burden, particularly related to the musculoskeletal pain associated with long feeding sessions, and the financial strain, primarily because of caregivers leaving employment to provide full-time care, reflect a broader theme of caregiver burden (Dambi et al., 2015; Sleigh, 2005; Taylor et al., 2021). Financial strain was compounded by costs for specialised foods and transport, emphasising the heightened difficulty in LMICs where resources are limited.

Participants identified a need for information and education from healthcare professionals on how to manage their child’s FSD, in the form of paper-based documents and practical training and coaching. This highlights the importance of interventions that enhance caregiver competence and confidence in providing care, as a means of reducing caregiver burden and increasing coping ability (Reinhard et al., 2008). The caregivers also reported a need for social support in the form of support groups and counselling, particularly mother-to-mother support groups. Caregivers perceived specific value in connecting with other mothers caring for children with CP and FSD, as they may benefit from the understanding and validation not found in other groups. Psycho-educational interventions that make the caregiver the main recipient of support may help to reduce burden and improve QoL (Reinhard et al., 2008). These findings are significant as they indicate that while the burden faced by caregivers of children with FSD has been recognised in the literature, it has not resulted in a shift in management approaches.

The impact of the COVID-19 pandemic exacerbated these challenges by increasing caregivers’ responsibilities and limiting access to healthcare and emotional support. Healthcare systems need to develop remote and accessible interventions to support caregivers during crises (McKinney et al., 2021). Despite these adversities, caregivers displayed resilience through coping strategies, balancing both problem- and emotion-focused approaches to manage the caregiving burden.

Finally, while caregiving was associated with significant challenges, the theme *Hopeful caregiving* revealed that caregivers also found purpose and fulfilment in their role, contributing to positive QoL outcomes. This reflects findings from previous research that caregiving can lead to personal growth and redefined priorities (Bailey & Harrist, 2017; Cohen et al., 2002).

### Limitations

While this qualitative study offers rich insights, it also has limitations. The purposive sampling technique and case study design limit the ability to generalise findings to a broader caregiver population. Additionally, because of COVID-19 restrictions, data collection shifted from focus groups to telephonic interviews, which, while safe, meant that participants’ nonverbal cues could not be observed and rapport building was reduced.

### Clinical implications

The findings underscore the importance of incorporating caregivers’ experiences in healthcare research to understand the impact of caregiving on their QoL and to guide the development of sustainable interventions. Healthcare professionals, particularly speech-language therapists, should consider combined interventions that address both the child’s feeding needs and the caregiver’s well-being. A family-centred approach is essential, offering culturally relevant materials and access to appropriate support networks. In LMICs, socioeconomic barriers may hinder the effectiveness of support services, suggesting a need for collaboration between healthcare professionals, caregivers, government bodies and non-government organisations. The insights from this study, particularly in the context of LMICs like South Africa, are valuable for informing targeted interventions aimed at improving the caregiving experience. Future studies could further build on these findings to enhance support for caregivers of children with CP and FSD.

## Conclusion

Caregiver burden and QoL are intertwined and shaped by the complex emotional, physical, social and financial demands of caregiving. Caregivers expressed a range of unmet support needs, highlighting the need for targeted, context-specific interventions to improve their well-being. Despite the challenges, caregivers also identified positive aspects of caregiving, including a sense of purpose and connection with their child. This study contributes valuable insights into caregiver experiences in a LMIC context.
